# Motorcycle crashes and upper extremity trauma

**DOI:** 10.1051/sicotj/2021007

**Published:** 2021-03-08

**Authors:** Erin Cravez, Kelsey A. Rankin, Nathaniel Ondeck, Lee Yaari, Michael Leslie, Carrie Swigart, Daniel H. Wiznia

**Affiliations:** 1 Yale School of Medicine 333 Cedar Street New Haven CT 06510 USA; 2 Hospital for Special Surgery 535 E 70th Street New York NY 10021 USA; 3 Arthroscopy and Sports Injuries Unit, Hasharon Hospital, Rabin Medical Center 7 Keren Kayemet Street 49372 Petach Tikva Israel; 4 Orthopedic Surgery Department, Hasharon Hospital, Rabin Medical Center 7 Keren Kayemet Street 49372 Petach Tikva Israel; 5 Affiliated with the Sackler School of Medicine, Tel Aviv University Ramat Aviv 69978 Tel Aviv Israel

**Keywords:** Motorcycle, Trauma, Helmet, Upper extremity, Intoxication

## Abstract

*Objectives*: Upper extremity injuries following motorcycle crashes (MCC) incur increased healthcare costs and rehabilitation needs. We aim to characterize the epidemiology of MCC upper extremity injuries and identify factors that influence the severity of and cost of care for upper extremity injuries. *Methods*: We performed a retrospective cohort analysis of 571 patients with upper extremity injuries after MCC at a level 1 trauma center from 2002 to 2013. We collected data pertaining to demographics, helmet use, toxicology, bony injury, Injury Severity Score (ISS), Glasgow Coma Scale (GCS), hospital length of stay (LOS), and cost. Continuous variables were compared using *t*-test or Wilcoxon rank test, depending on data distribution, and dichotomous variables were compared using Pearson’s chi-squared or Fisher’s exact tests. Regression models were used to evaluate the effect of intoxication or helmets on injury location, severity, cost of care, and LOS. *Results*: The incidence of MCC upper extremity injury was 47.5%, with hand and forearm fractures the most common injuries (25.5% and 24.7% of total injuries). Intoxicated patients were more likely to have a high cost of care (*p* = 0.012), extended LOS (*p* = 0.038), plastic surgery involvement in their care (*p* = 0.038), but fewer upper extremity bony injuries (*p* = 0.019). Non-helmeted patients sustained less upper extremity bony injuries (*p* < 0.001) and upper extremity soft tissue injuries (*p* = 0.001), yet more severe injuries (ISS ≥ 30, *p* = 0.006 and GCS < 9, *p* < 0.01) than helmeted patients. *Conclusion*: Upper extremity injuries are common in motorcyclists. Despite vital protection for the brain and maxillofacial injury, helmeted MCC patients have an increased incidence of upper extremity injuries compared to non-helmeted patients, but overall have less severe injuries. Intoxicated patients have fewer upper extremity bony injuries, but the higher cost of care, and extended LOS. Therefore, even with the increased risk of injury helmets may expose to the upper extremity, helmets reduced overall morbidity and mortality. In addition to mandatory helmet laws, we advocate for further development of safety equipment focusing specifically on the prevention of upper extremity injuries.

## Introduction

Motorcycle crash (MCC) injuries are a global epidemic. The incidence of MCC accidents is disproportionally high when compared with other road vehicles. For example, at one urban trauma center, MCCs generated 40% of all road traffic accident trauma patients, despite the fact that motorcycles comprised less than 10% of road vehicles in the country [1]. MCCs are also associated with significantly increased morbidity and mortality [2]. A recent report by the National Highway Traffic Safety found that motorcyclists have a 29 times higher risk of mortality per mile than occupants of enclosed vehicles [3]. Survivors of MCCs suffer significant short- and long-term morbidity from these injuries, placing a significant economic burden on the individual and the healthcare system, as displayed by lost wages, unemployment, and long-term medical expenses [4, 5].

Upper extremity injuries are a significant portion of MCC trauma, but a focus on these injuries is lacking in the literature. To our knowledge, there is only one significant reference in the literature [6]. The study by Paryavi et al. found that upper extremity injuries are quite common, constituting 35% of all motorcycle injuries [6]. They also found that patients who suffered upper extremity injuries might experience lower mortality and increased needs for rehabilitation services after discharge [6]. However, this study did not address the epidemiology of these injuries in depth. They established a need for characterization of upper extremity injury patterns, an understanding of the impact of protective measures and motorcycle drivers’ behavior such as intoxicated driving, and cost analysis to help inform injury prevention, awareness, and resource allocation.

Therefore, the goal of this study was to address previous gaps in this field, characterize upper extremity bony and soft tissue injuries occurring in MCCs, and examine the clinical and economic impact of helmet use and intoxication on MCC patients with upper extremity injuries. We chose variables shown to influence injury severity and accident patterns [7–9]. We hypothesized that non-helmeted and intoxicated patients would suffer significantly more severe injuries, more bony and soft tissue injuries, and increased treatment costs.

## Materials and methods

### Data collection

This is a retrospective cohort study evaluating motorcycle trauma data at a level 1 trauma center. The hospital trauma registry was queried for data from July 2, 2002 to December 31, 2013. The study included any patient who activated the trauma system (full and modified), had an International Classification of Diseases, Ninth Revision (CDC 2013; ICD-9) code between 800 and 959.99, or alerted a general trauma consult after an MCC, and was diagnosed with an upper extremity injury at the shoulder level or distally. Patients were then stratified to alcohol intoxicated and non-intoxicated and to helmeted and non-helmeted cohorts. “Intoxicated” was defined as any blood alcohol concentration (BAC) > 0, and “sober” was defined as BAC = 0. For the purposes of this study, we define “intoxicated” as the consumption of any alcohol (BAC > 0), not legal intoxication while driving.

Imaging of all patients was independently reviewed by three of the authors, including review of radiology reports and confirmation by an independent review of plain radiographs. Patients who had radiographic imaging inconsistent with their ICD-9 had their diagnoses revised or were removed from the study if they had no upper extremity injury.

Patient variables collected included demographic information (age, gender, and race), helmet use, blood alcohol concentration (BAC), Injury Severity Score (ISS), Glasgow Coma Scale (GCS), hospital length of stay (LOS), number of surgical procedures, and total hospital costs. Injuries to the upper extremity were divided into fractures of the upper, midshaft, and distal humerus; upper, midshaft, and distal forearm; and hand according to the AO Fracture Classification [10]. Information on concomitant injuries such as dislocations, amputations, arterial injury, compartment syndrome, soft tissue injury, and the need for plastic surgical intervention was collected. Patients with bilateral or multiple upper extremity injuries were recorded such that each bony fracture was reported as a separate injury.

Data on costs directly attributed to caring for the patient was collected. These costs, called direct hospital costs, include labor, supplies, medications, diagnostic tests, and surgery. Insurance information was obtained from the institution’s Decision Support Office. Costs were adjusted to 2017 dollars, using the Bureau of Labor Statistics Medical Consumer Price Index. Hospital charges were calculated from the direct hospital costs using the hospital’s cost-to-charge ratio.

### Statistics

Independent sample *t*-tests or non-parametric Wilcoxon tests were used to analyze continuous variables, depending on data distribution. Normality was assessed using the Shapiro-Wilk test. Pearson’s chi-squared or Fisher’s exact tests were utilized for dichotomous variables. Univariate logistic regression models were used to assess the association of helmet use and intoxicated driving on injury patterns such as the location of the injury, injury severity, bony injuries, soft tissue injuries, plastics involvement in care, hospital LOS, and cost of care. Multivariate regression analyses were conducted to evaluate the effect of behavioral characteristics on injury severity, bony and soft tissue injury, plastics involvement, hospital LOS, and cost of care. We controlled for confounders on the relationship between patients’ behavior and injury severity, injury characteristics and hospital LOS, and costs, based on those that were significantly different between helmeted vs. non-helmeted, and between intoxicated vs. non-intoxicated. These included intoxication status, helmet use, age, sex, and race. Variables that were intermediate to helmet use, ISS, or GCS were not included. For LOS and cost of care analyses, subgroup analysis was utilized to identify patients with the highest-burden on total hospital costs. These were identified as patients within the upper quartile of the length of stay (≥75th percentile: 11 days) and adjusted hospital charges (≥75th percentile: 21,799.83 USD), and univariate logistic regression models were performed to assess the association of helmet use and intoxicated driving on this subgroup.

Statistical testing was performed two-tailed. Calculations were completed using IBM SPSS version 21 (IBM, Armonk, New York). Significance was set at *p* < 0.05. This study was approved by the Human Research Protection Program at our institute, HIC# 1403013641.

## Results

### MCC patient injuries

From July 2, 2002 to December 31, 2013, a total of 37,086 patients were entered into the trauma registry, of which 1,066 patients (2.9%) were involved in an MCC. Five hundred seventy-one (53.6%) of these MCC patients sustained at least one upper extremity injury, either bony or soft tissue, with a total of 338 upper extremity fractures in 271 patients (47.5%). Patients were predominantly young (38.4 ± 13.6 years), male (89.3%), and White (76.7%) (Table 1).

**Table 1:** Demographics and injury characteristics of patients involved in an MCC with an upper extremity injury.

	Number (#)	Percent (%)
Total patients	571	100.00
Age (years); mean ± *SD*	38.48 ± 13.62	
0–19	35	6.13
20–29	153	26.80
30–39	107	18.74
40–49	141	24.69
50–59	102	17.86
60–69	26	4.55
≥ 70	5	0.88
Unknown	2	0.35
Sex		
Male	508	89.28
Female	61	10.72
Race/ethnicity		
White	438	76.71
Black	61	10.68
Hispanic	57	9.98
Other/unknown	15	2.63
Helmet usage		
Helmeted	158	27.67
Non-helmeted	383	67.08
Unknown	30	5.25
EtOH intoxication		
Intoxicated (BAC > 0)	189	33.10
Sober (BAC = 0)	359	62.87
Unknown	23	4.03
GCS; mean ± *SD*	12.78 ± 4.35	
Minor (≥13)	422	73.91
Moderate (9–12)	11	1.93
Severe (<9)	92	16.11
Unknown	46	8.06
ISS; mean ± *SD*	18.26 ± 13.04	
< 15	286	50.09
15–19	73	12.78
20–24	88	15.41
25–29	53	9.28
≥ 30	69	12.08
Unknown	2	0.35
Any bony injury		
Patients	271	47.46
Fractures*	338	59.19
Humerus fracture	53	15.68
Forearm fracture	141	41.72
Hand fracture	144	42.60
Upper extremity dislocation	88	15.41
Acromioclavicular (AC)	20	22.73
Shoulder	18	20.45
Wrist	15	17.05
Other	35	39.77
Any soft tissue injury	36	6.30
Compartment syndrome	14	2.45
Amputation	6	1.05
Arterial injuries	3	0.53
Plastics involvement in care	43	7.53

* Individual fractures of patients sustaining bilateral or multiple upper extremity fractures are included as separate injuries.

MCC: motorcycle crash; EtOH: alcohol; BAC: blood alcohol concentration; *SD*: standard deviation.

The majority of patients with upper extremity injuries were non-helmeted (67.1%) and sober (63.9%). Patients had a mean Glasgow Coma Scale (GCS) of 12.8 ± 4.4 and a mean Injury Severity Score (ISS) of 18.3 ± 13.0. When looking at the distribution of fractures, 271 (47.5%) motorcyclists sustained 338 upper extremity fractures, 15.7% of which were humeral, 41.7% were forearm, and 42.6% were hand fractures. 15.4% of patients had an upper extremity joint dislocation, of which acromioclavicular (AC) joint dislocations were the most common (22.7%) (Table 1). Soft tissue injuries were less commonly documented, with 6.3% of patients having any soft tissue injury (Table 1).

To understand the hospital burden of upper extremity injuries from MCC, we next analyzed LOS and direct patient costs. The mean LOS for patients was 9.4 ± 12.6 days. Patients with the highest-burden, identified as those in the top quartile (75th percentile), were those with mean LOS > 11. Mean direct costs, which included labor, supplies, medications, diagnostic tests, and surgery, was 18,372.66 ± 26,667.78 USD/patient. Patients with the highest-burden, identified as those in the top quartile (75th percentile), were those with mean cost > 21,799.83 USD/patient.

### Helmet use and upper extremity injuries

We next looked at the association between helmet use and the location of upper extremity fracture (Table 2). We found no differences in the location of the upper extremity fracture between helmeted vs. non-helmeted groups (*p* = 0.408). Next, we conducted subgroup analyses of two groups: humerus and forearm. When looking at helmet use and humeral fracture location, we found that non-helmeted patients sustained more proximal humerus fractures than helmeted patients (*p* < 0.005). We found no differences between helmeted vs. non-helmeted groups in forearm fracture location (*p* = 0.283) (Table 2).

**Table 2:** Impact of helmet usage on distribution of upper extremity fractures.

	Helmeted (*n* = 142); *n* (%)	Non-helmeted (*n* = 171); *n* (%)	*p*-value
Location			
Humerus	19 (13.38%)	30 (17.54%)	0.408
Forearm	55 (38.73%)	77 (45.03%)	
Hand	68 (47.89%)	64 (37.43%)	
Humerus			
Proximal	5 (26.32)	14 (46.67%)	**<0.005**
Shaft	7 (36.84%)	9 (30.00%)	
Distal	7 (36.84%)	7 (23.33%)	
Forearm			
Proximal	12 (21.82%)	12 (15.58%)	0.283
Shaft	19 (34.55%)	22 (28.57%)	
Distal	24 (43.64%)	43 (55.84%)	

Bolded values indicate statistical significance. Significance was set at *p* < 0.05.

Next, we analyzed the relationship between helmet use and a range of adverse outcomes (Table 3). Non-helmeted patients arrived at the hospital with more severe injuries, as characterized by ISS ≥ 30 (*p* = 0.007) or GCS < 9 (*p* = 0.006). Helmeted patients were significantly more likely to have sustained an upper extremity bony injury (*p* < 0.001) or soft tissue injury (*p* = 0.001). We found no differences based on helmet status when looking at plastic surgery involvement (*p* = 0.971), LOS (*p* = 0.859), top quartile for LOS (*p* = 0.320), direct hospital cost (*p* = 0.720), or top quartile for direct hospital cost (*p* = 0.755) (Table 3).

**Table 3:** Impact of helmet usage and intoxication on selected adverse outcomes.

	Helmet usage			Intoxication		
Adverse outcome	Helmeted (*n* = 158); *n* (%)	Non-helmeted (*n* = 383); *n* (%)	*p*-value	Sober (*n* = 359); *n* (%)	Intoxicated (*n* = 189); *n* (%)	*p*-value
Severe GCS (<9)	15 (9.49%)	73 (19.06%)	**0.006**	54 (15.04%)	36 (19.05%)	0.229
ISS ≥ 30	10 (6.33%)	56 (14.62%)	**0.007**	44 (12.26%)	23 (12.17%)	0.976
Any bony injury	101 (63.92%)	151 (39.43%)	**<0.001**	182 (50.70%)	76 (40.21%)	**0.019**
Any soft tissue	18 (11.39%)	14 (3.66%)	**0.001**	20 (5.57%)	15 (7.94%)	0.282
Plastic surgery involvement	11 (6.96%)	27 (7.05%)	0.971	21 (5.85%)	20 (10.58%)	**0.045**
Extended LOS (>75th percentile)	36 (22.78%)	103 (26.89%)	0.320	83 (23.18%)	59 (31.38%)	**0.038**
High cost (>75th percentile)	38 (24.05%)	97 (25.33%)	0.755	74 (21.70%)	58 (31.69%)	**0.012**

GCS: Glasgow coma scale; ISS: injury severity score; LOS: length of stay (days). Sober: blood alcohol concentration (BAC) = 0; intoxicated: BAC > 0. Significance set at *p* < 0.05.

### Alcohol intoxication and upper extremity injuries

We next sought to understand the relationship between alcohol intoxication and upper extremity injuries (Table 3). Intoxicated patients were significantly more likely to require plastic surgery intervention (*p* = 0.045), have extended LOS (*p* = 0.038), and higher cost of care (mean: *p* = 0.019; extended: *p* = 0.012). Sober patients were more likely to have sustained any upper extremity bony injury (*p* = 0.019). We saw no differences based on intoxication status when looking at injury severity (*p* = 0.976), GCS score (*p* = 0.229), or upper extremity soft tissue injury (*p* = 0.282) (Table 3).

### Multivariable analysis: helmets and intoxication on upper extremity injuries

To further understand the interaction of helmets and intoxication status on upper extremity injury, we performed multivariate analyses, controlling for all significant variables from univariate analyses (Tables 2 and 3). We found that non-helmeted patients arrive at the hospital with more severe injuries (GCS < 9 odds ratio (OR) = 2.28 [1.24–4.17]; *p* = 0.008 or ISS ≥ 30 OR = 2.42 [1.18–4.93]; *p* = 0.015), whereas intoxication status had no impact on either GCS < 9 (OR = 1.28 [0.79–2.08]; *p* = 0.322) or ISS ≥ 30 (OR = 0.91 [0.52–1.50]; *p* = 0.752). Helmeted patients and intoxicated patients were less likely to sustain a bony injury (OR = 0.38 [0.25–0.57]; *p* < 0.001 and OR = 0.019 [0.43–0.93]; *p* = 0.019, respectively). Intoxicated patients were more likely to have a top quartile cost/patient (OR = 1.62 [1.07–2.45]; *p* = 0.024), whereas helmet status had no impact on top quartile cost (OR = 0.99 [0.63–1.55]; *p* = 0.953) (Table 4).

**Table 4:** Impact of helmet and intoxication status on selected adverse outcomes.

	Non-helmeted (*n* = 383)		Intoxicated (*n* = 189)	
Adverse outcome	OR [CI]	*p*-value	OR [CI]	*p*-value
Severe GCS (<9)	2.28 [1.24–4.17]	**0.008**	1.28 [0.79–2.08]	0.322
ISS ≥ 30	2.42 [1.18–4.93]	**0.015**	0.91 [0.52–1.60]	0.752
Any bony injury	0.38 [0.25–0.57]	**<0.001**	0.02 [0.43–0.93]	**0.019**
Any soft tissue injury	0.57 [0.20–1.60]	0.288	1.21 [0.42–3.47]	0.726
Plastic surgery involvement	0.83 [0.39–1.78]	0.638	1.89 [0.94–3.79]	0.074
Extended LOS (>75th percentile)	1.13 [0.72–1.78]	0.587	1.43 [0.95–2.17]	0.088
High cost (>75th percentile)	0.99 [0.63–1.55]	0.953	1.62 [1.07–2.45]	**0.024**

OR: odds ratio; CI: 95% confidence interval; GCS: Glasgow coma scale; ISS: injury severity score; LOS: length of stay (days). Controlled for age, sex, and race. For each outcome, helmet status and intoxication status were included in the model. Significance set at *p* < 0.05.

## Discussion

MCCs represent a significant portion of all road traffic accidents, despite the low proportion of motorcycles on the road [1]. Importantly, they are also highly associated with morbidity and mortality: motorcyclists have a 29 times higher risk of mortality per mile than occupants of enclosed vehicles [3]. Despite representing a significant burden of MCC trauma, research into upper extremity injuries resulting from MCCs is lacking: we found only one such study, which characterized the need for rehabilitation and mortality risk in those experiencing upper extremity injuries resulting from MCCs [6]. Therefore, we sought to address existing gaps in this literature and characterize upper extremity injury patterns and driver protective/risk behaviors.

Limitations of our study include under-representation of non-intoxicated and helmeted patients in our cohort. This study only includes patients with injuries severe enough to warrant transport to a level 1 trauma center. Compared to non-helmeted patients, fewer helmeted patients are hospitalized after an accident [11]. Also, both intoxicated and non-helmeted individuals are more likely to expire at the accident scene [12]. Our analysis excluded patients who died prior to arrival or on initial resuscitation in the trauma bay. Collectively, we may have missed a number of patients. This selection bias may help explain our results demonstrating fewer upper extremity injuries among intoxicated and non-helmeted individuals. As our focus was the upper limb, defined by the shoulder distally, we did not include other injuries of the comprehensive shoulder girdle such as the scapula or clavicle. A focused analysis of these injuries may prove a potential avenue for future study. Our study was also limited by its retrospective nature (2002–2013), precluding a complete investigation of the effect of other potential confounders. One potential cofounder of note is other protective equipment, such as motorcycle jackets. Data were obtained from an inpatient registry, which did not include information on long-term and functional outcomes. Post-hospitalization costs such as post-discharge rehabilitation lost wages due to disability, and loss of productivity was not included. Prior studies have reported that a higher proportion of non-helmeted patients need institutional care after discharge, which could increase the cost of long-term care and lead to a significant difference between groups [13]. We were also limited by data collected at a single institution; however, our results are consistent with previous studies regarding motorcycle injury cost conducted at a population level in the state of Connecticut [14, 15].

The incidence of upper extremity injuries in motorcyclists in this population was high (47.5%). This finding is in accordance with an India-based study by Fitzharris et al*.* (46.0%), although is relatively high in comparison with a study based at the University of Maryland (35.0%) [6, 16]. We can account for this difference, as soft tissue injuries such as lacerations, skin de-gloving, and injuries requiring plastic intervention were not included in these studies. This reflects the need for further exploration of upper extremity injuries in MCC crashes. 89.3% of patients in our study were men, similar to other published studies on MCC injuries and is likely related to a greater incidence of motorcycle use among men [6, 17–19].

Forearm and hand fractures were the most common upper extremity injuries. This is consistent with previous studies examining upper extremity injuries in MCC patients [6, 17]. There was a trend of injuries clustering around the proximal humerus and distal forearm. However, this was not found to be statistically significant. This distribution may be related to the position of the motorcyclist, with the flexed elbows furthest from impact, or the patient’s helmet status. We found that helmeted patients are significantly less likely to have sustained a proximal humerus fracture, but were more likely overall to have any bony or soft tissue injuries. This could suggest a protective effect of helmets against shoulder injury. Although this mechanism is unclear, it is possible that energy transmission through the helmet and neck flexion at the time of injury may shield the proximal humerus and shoulder girdle from injury. It is also possible that the patient’s helmet status could influence both positionings on the motorcycle as well as fall patterns, based on an unconscious understanding of protection – or lack thereof – of distinct vital parts of the body. This may account for the increased representation of soft tissue and bony injuries in helmeted patients. Another possible explanation is an increase in high-risk behaviors: perhaps helmeted patients engage in risk compensation [20], exhibiting more dangerous driving behaviors due to their perceived protection, which could account for the increased likelihood of bony and soft tissue injuries. We believe this correlation merits further follow-up.

We examined upper extremity dislocations among patients in our cohort and found that AC joint, shoulder, and wrist dislocations were the most common types of dislocation. Although previous studies have commented on a similar incidence of shoulder and wrist dislocations, our work is the first to report the incidence of AC joint dislocations among MCC patients, which was the most common upper extremity dislocation (22.7%) [6]. Soft tissue injuries were relatively infrequent in our study (6.3% of patients). 2.5% of patients suffered from compartment syndrome, 1.1% from upper extremity amputation, and 0.5% from an arterial injury. This may have been limited by poor identification or documentation of soft tissue injuries relative to bony injury. Although infrequent, compartment syndrome is limb-threatening in high-energy trauma and has not previously been investigated among MCC patients. Given these findings, we recommend that future studies address AC joint dislocations, the shoulder girdle complex, and compartment syndrome when examining the epidemiology and outcomes of MCC patients.

The influence of patients’ behavior on outcomes, specifically helmet use and intoxication, has been previously investigated in the literature [21, 22]. Helmet use has been associated with decreased injury severity, increased survival, and increased need for rehabilitation after MCC [6, 22]. Our findings support the existing literature with non-helmeted patients having more severe injury scores. However, non-helmeted patients were also less likely to sustain upper extremity bony or soft tissue injuries overall, but have more severe injuries overall. Remarkably, the effect of helmets did not translate to lower costs of care, despite prior literature that helmet laws decrease medical costs [23]. We analyzed both differences in overall cost (*p* = 0.720), as well as differences in high cost (>75th percentile; *p* = 0.755). Nevertheless, we did not find a statistically significant difference in cost of care between helmeted and non-helmeted individuals using this method. Additionally, there were no observed differences in LOS associated with wearing a helmet.

Intoxication was associated with significantly higher hospital costs, even after controlling for helmets status. Previous studies have identified non-helmeted riders as more likely to engage in riskier behaviors, such as driving while intoxicated, and higher rates of alcohol use in non-helmeted patients are often cited as a confounder that explains worse clinical outcomes [22, 24]. However, after adjusting for intoxication status, we did not find that helmets ultimately affected the cost of care, suggesting that intoxication status is the primary driver for this increase in the cost of care.

In conclusion, given the scarcity of information on upper extremity injuries in motorcycle crashes, our study of a large cohort of motorcyclists with upper extremity injuries provides valuable insight into the epidemiology and potential outcomes of these accidents. We have demonstrated that upper extremity injuries are common in motorcyclists. Individuals with upper extremity injuries who are not intoxicated and wear a protective helmet arrive at the hospital with less severe injuries overall. Non-intoxicated patients are also hospitalized for shorter periods of time and accumulate a lower cost of care. Helmeted MCC patients have an increased incidence of upper extremity injuries compared to non-helmeted patients but overall have less severe injuries.

## Conflict of interest

The authors declare that there are no conflicts of interest.

## Funding

This research received no specific grant from any funding agency in the public, commercial, or not-for-profit sectors.

## Research ethics and patient consent

This article does not contain any studies with human participants or animals performed by any of the authors.
